# Critical Role of the Disintegrin Metalloprotease ADAM-like Decysin-1 [ADAMDEC1] for Intestinal Immunity and Inflammation

**DOI:** 10.1093/ecco-jcc/jjw111

**Published:** 2016-05-25

**Authors:** Nuala R. O’Shea, Thean S. Chew, Jenny Dunne, Rebecca Marnane, Bahman Nedjat-Shokouhi, Philip J. Smith, Stuart L. Bloom, Andrew M. Smith, Anthony W. Segal

**Affiliations:** ^a^Division of Medicine, University College London, London, UK; ^b^Department of Gastroenterology, University College London Hospital, UK; ^c^Microbial Diseases, Eastman Dental Institute, University College London, London, UK

**Keywords:** *Citrobacter rodentium*, colitis, DSS

## Abstract

**Background and Aims:**

ADAM [A Disintegrin And Metalloproteinase] is a family of peptidase proteins which have diverse roles in tissue homeostasis and immunity. Here, we study ADAM-like DECysin-1 [ADAMDEC1] a unique member of the ADAM family. ADAMDEC1 expression is restricted to the macrophage/dendritic cell populations of the gastrointestinal tract and secondary lymphoid tissue. The biological function of ADAMDEC1 is unknown but it has been hypothesised to play a role in immunity. The identification of reduced ADAMDEC1 expression in Crohn’s disease patients has provided evidence of a potential role in bowel inflammation.

**Methods:**

*Adamdec1*^*-/*-^ mice were exposed to dextran sodium sulphate or infected orally with *Citrobacter rodentium* or *Salmonella typhimurium*. The clinical response was monitored.

**Results:**

The loss of *Adamdec1* rendered mice more susceptible to the induction of bacterial and chemical induced colitis, as evidenced by increased neutrophil infiltration, greater IL-6 and IL-1β secretion, more weight loss and increased mortality. In the absence of *Adamdec1, g*reater numbers of *Citrobacter rodentium* were found in the spleen, suggestive of a breakdown in mucosal immunity which resulted in bacteraemia.

**Conclusion:**

In summary, ADAMDEC1 protects the bowel from chemical and bacterial insults, failure of which may predispose to Crohn’s disease.

## 1. Introduction

ADAM proteins are related to matrix metalloproteases and snake venom proteins.^1^ In recent years, ADAMs have become increasingly recognised as important in inflammation and tissue repair, and a growing interest has developed in the potential role of metalloproteases in gut homeostasis and bowel inflammation.^2^ ADAMDEC1, first identified in 1997, is the sole member of a subsidiary class of the ADAM family.^1^ It has been linked to a number of inflammatory diseases including atherosclerosis,^3^ pulmonary sarcoidosis,^4^ osteoarthritis,^5,6^ Crohn’s disease,^7,8^ and to gastrointestinal [GI] malignancies–gastric adenocarcinoma^9, 10^ and colorectal cancer.^11, 12^ The physiological role of ADAMDEC1 remains to be determined.

ADAMDEC1 is a unique member of the ADAM family: it contains pro- and catalytic domains but has a truncated disintegrin domain and lacks the typical transmembrane domain and cytoplasmic tail.^13^ It is believed to be a soluble, secreted protein and has recently been identified in osteoarthritis synovial fluid^5^ and in the supernatant from un-stimulated ADAMDEC1-transfected HEK293 cells.^14^ It is predicted that the disintegrin site is non-functional as the ‘disintegrin loop’, reported to be essential for integrin binding,^13^ is missing. The other distinguishing feature of ADAMDEC1 is that it is the only mammalian ADAM protease in which a histidine [H] is replaced by an aspartic acid [D] residue within the zinc-binding sequence of the metalloprotease domain, *H*EXX*H*XXGXX*D*, [H362D]. The metalloprotease site of ADAMDEC1 has been shown to be proteolytically active *in vitro* but the natural ligands remain unclear.^14^ Recently it has been demonstrated that the unique nature of the active site in ADAMDEC1 allows it to escape inhibition by the tissue inhibitors of metalloproteases 1–3 [TIMP1-3].^15^ ADAMDEC1 seems to have evolved to function independently from the normal intrinsic inhibitory mechanisms that regulate other metalloproteases.

The tissue distribution of ADAMDEC1 in the non-inflamed, steady state is almost exclusively in the GI tract and to a lesser extent in lymphoid tissue and spleen in humans.^16^ At a cellular level, ADAMDEC1 expression has been identified in macrophages isolated from non-inflamed human intestine^16^ and in mature CD40 activated CD11c^+^ dendritic cells from the thymus and tonsils.^17,18^ Although undetectable in monocytes*, in vitro* it is upregulated when these cells mature to macrophages and more rapidly if this occurs in the presence of lipopolysaccharide [LPS] or 1α, 25-dihydroxy vitamin D_3._^16^ This responsiveness to LPS and the association with a number of inflammatory diseases have resulted in speculation that ADAMDEC1 has an active role in the immune system, but no conclusive evidence has been presented to date.

We have used three different bowel inflammation models, dextran sodium sulphate [DSS], *Citrobacter rodentium [C. rodentium]*, and *Salmonella typhimurium [S. typhimurium]* in *Adamdec1*-deficient mice to determine whether the lack of this molecule alters the susceptibility or immune response to these insults. We have demonstrated that in the absence of *Adamdec1*, mice are more susceptible to infection and colitis. These findings suggest a physiological role for ADAMDEC1 in protecting the GI tract against infection and inflammation.

## 2. Materials and Methods

### 2.1. *Adamdec1^+/+^* and *Adamdec1^-/-^* mice

Animal studies were performed in accordance with the UK Animals [Scientific Procedures] Act 1986 and European Directive 2010/63/EU on the protection of animals used for scientific purposes. *Adamdec1^+/-^* mice were generated by targeted mutagenesis of the *Adamdec1* gene 1227 on chromosome 8 and insertion of a neomycin-resistant cassette into exon 11. The line was reconstituted from frozen embryos from the Deltagen repository. Embryonic stem cells were re-derived from 129/OlaHsd mice. The chimeric mice were back-crossed onto C57BL/6 mice [Charles Rivers] for a minimum of 6 generations. *Adamdec1*^*+/+*^, *Adamdec1^-/-^*, and C57BL/6 wild-type mice were bred and maintained in specific pathogen-free [SPF] cages in the Biological Sciences Unit, UCL. Genotyping was performed by polymerase chain reaction [PCR] using *Adamdec1* wild-type gene-specific primers: AGCTTGAGCGCAAACCCAATGCTTC and CCTCAGGTACTGATTCATCACACAG, 322bp, and *Adamdec1*^*-/*-^ mice primers: GACGAGTTCTTCTGAGGGGATCGATC and CCTCAGGTACTGATTCATCACACAG, 600bp.

#### 2.1.1. Dextran sodium sulphate-induced colitis

Mice at 9–12 weeks old were administered drinking water containing 2% DSS [MW 36 000–50 000] [MP Biomedicals, Cambridge, UK] for 7 days as previously described.^41^ Mice were weighed daily and sacrificed between 0 and 21 days for tissue and blood collection.

#### 2.1.2. Bacterial induced colitis

*C. rodentium* strain ICC169 was kindly provided by Gad Frankel, Imperial College London, UK. *C. rodentium* was prepared and administered as previously described.^42^ *Adamdec1*^*-/*-^ and *Adamdec1*^*+/+*^ mice at 9–12 weeks old were orally gavaged 10^8^ or 10^9^ colony-forming units [CFU] of *C. rodentium*.

Mice were weighed daily. Faeces and tail bleeds were collected for *C. rodentium* culture and serum cytokine analysis. At set time points between 0 and 13 days, mice were culled. Blood from tail bleed or cardiac puncture, colons, and spleens were collected. Blood, disrupted spleens, and dispersed faeces in phosphate buffered saline [PBS] were plated on LB agar plates containing 50 µg/ml nalidixic acid, to quantify the *C. rodentium*.

*S. enterica* serovar *Typhimurium* [JT11] was kindly provided by Dr Elizabeth de Pinna, Public Health England, UK. *S. typhimurium* was cultured overnight, for 16h, in LB broth, centrifuged at 4 000g, washed and resuspended in sterile PBS. Mice at 9-12 weeks old were pretreated with metronidazole [0.75g/l diluted in drinking water] for 5 days, before restarting drinking water for 20h, followed by a 4-h fast, before oral gavage with 10^8^ *S. typhimurium*. Mice were then monitored and weighed for 48h.

### 2.2. Murine large bowel lamina propria cell isolation

Colonic lamina propria cells were isolated as previously described.^42^

#### 2.2.1. Flow cytometry

Murine colonic lamina propria cells were incubated with LIVE/DEAD^®^ stain [Invitrogen L23105], blocked in CD16/CD32 Fc block [eBioscience 16–0161] before staining with CD45 PerCP-Cy™5.5 [BD 550994], CD11b V450 [BD 560455], CD11c APC [BioLegend 117309], Gr1 PE [BD 553128] [eBioscience 11–4801], and CD3 PE-Cy^TM^7 [BD 560591] antibodies, then fixed in 1% formaldehyde. Cells run on a BD LSR Fortessa [BD Biosciences, Oxford, UK] were analysed using FlowJo 7.6.4 [Tree Star, Inc].

#### 2.2.2. Quantitative reverse transcription PCR

Intestinal samples [5 mm] were homogenised in RNA*later* [Qiagen] using the TissueLyser LT [Qiagen]. RNA was harvested using the RNeasy^®^ Mini kit [Qiagen]. Total RNA was converted to complementary DNA [cDNA] using the QuantiTect^®^ Reverse Transcription Kit [Qiagen]. Quantitative reverse transcription PCR [qRT-PCR] of mouse *Adamdec1* was performed using the QuantiFast SYBR^®^ Green PCR kit [Qiagen], in duplicate on a Mastercycler^®^ ep *realplex* [Eppendorf] [Forward primer: GTAATTGAGGCTAAAAAAAAGAATAATGTG, reverse primer: GCGTGGCCCAACTCATG]. Normalised mean gene expression values ± standard deviation [SD] were determined from duplicate cycle threshold [Ct] values for each gene and the housekeeping gene peptidylprolyl isomerase A [*Ppia*]. Relative transcript levels were determined by the 2−ΔΔCt method.^43^]

### 2.3. *In situ* hybridisation

Predesigned *in situ* probes were purchased from Source Bioscience [human ADAMDEC1 No 2402230 and mouse Adamdec1 No 1511966], cloned into pT7T3D-PacI and expressed in One Shot® TOP10 Chemically Competent *E. coli* [Lifetechnologies, UK]. Probes were linearised with NotI / EcoRI and riboprobes generated using the Roche DIG RNA Labelling Kit [SP6/T7] [Sigma, UK]. Healthy human colonic and small bowel tissue was obtained from surgical resection margins from patients at University College London Hospital, UCLH [NHS National Research Ethics Project Number 10/H0806/115]. Specimens were immediately fixed in DepC-treated 4% PFA overnight followed by cryoprotection in DepC treated 20% sucrose in PBS overnight at 4ºC. Bowel was harvested from freshly culled C57BL/6 mice fixed and cryoprotected as above and tissue was rinsed and fixed in OCT; 20-µm sections were cut, mounted, and dried in a desiccator. For hybridisation, the riboprobes were diluted 1/1000 in hybridisation buffer [50% deionised formamide, 1 x Denhardt’s solution [Invitrogen], 10% dextran sulphate [Sigma], 0.1mg/ml yeast tRNA [Roche], and 1 x ‘salts’ [0.2M NaCl, 5mM EDTA, 10mM Tris-HCl, 5mM NaH2PO4.2H2O and 5mM Na2HPO]. Sections were incubated at 65ºC overnight in a humidified chamber. Slides were washed three times for 5min in MABT buffer [100mM Maleic Acid, 150mM NaCl and 0.1% Tween-20, pH 7.5] and twice for 30min at 65 ºC in 1xSSC, 50% formamide, and 0.1% Tween-20. Slides were rinsed for two 5-min washes in MABT buffer. Sections were blocked in 2% blocking reagent [Roche], 10% heat-inactivated sheep serum [GenTex] in MABT buffer for 1h at room temperature in a humidified chamber, then incubated with 1:1000 AP conjugated anti-DIG Fab fragments [Roche 1093274] diluted in 2% blocking reagent overnight at 4 ºC. Slides were washed three times for 10min in MABT buffer, then twice for 2min in GB3 buffer [100mM Tris, pH 9.8, 100mM NaCl and 50mM MgCl_2_]. Slides were incubated in developing buffer for nitroblue tetrazolium staining [100mM Tris pH 9.8, 100mM NaCl, 50mM MgCl_2_, 5% PVA, 0.11mM BCIP and 0.12mM NBT] for 6h at 37ºC, protected from light in a humidified chamber. Dehydrated slides were mounted in a xylene-based mountant [DPX].

#### 2.3.1. Anti-mouse ADAMDEC1 antibody

The catalytic domain of murine *Adamdec1* was cloned into a GB1-pBR22b expression construct and the protein was expressed in *E coli*. This GB1-ADAMDEC1 protein, with the solubility enhancer GB1 at the N-terminus and a His tag at the C-terminus, was confirmed by mass spectrometry [data not shown]. The protein was injected into rabbits to produce the anti-serum.

### 2.4. Histology and immunohistochemistry

Immunohistochemical staining of non-inflamed human intestinal tissue was performed by University College London Hospital [UCLH] histopathology department. Sections were preserved in formalin for 24h before paraffin embedding; 5-μm sections underwent automated dewaxing. Endogenous peroxidase was blocked using 3–4% [v/v] hydrogen peroxide. Sections were stained with monoclonal anti-ADAMDEC1 antibody, raised in mouse [clone 6C4 Sigma WH0027299M1], 1:100 dilution, for 30min at room temperature following heat-induced epitope retrieval for 20min using an EDTA-based [pH 9.0] epitope retrieval solution. Signal visualisation using the Bond Polymer Refine Detection kit [Leica Biosystems, Nussloch GmbH] with DAB Enhancer [Leica Biosystems] was performed on the Bond-III automated staining platform [Leica Biosystems]. Cell nuclei were counterstained with haematoxylin.

Mouse colonic tissue was fixed in 10% neutral buffered formalin [CellPath] overnight then paraffin-embedded using a Leica TP1050 tissue processor; 5-μm sections were cut, dewaxed, and endogenous peroxidase was blocked using 3–4% [v/v] hydrogen peroxide. Sections were stained, using a Leica ST4040 linear stainer, with VFM Harris’ haematoxylin [CellPath], differentiated in 0.2% acid alcohol and Eosin Y [VWR], and mounted in Pertex [Leica Biosystems] or blocked with 5% goat serum [Sigma G67G7] [diluted in distilled water] and stained with an anti-mouse ADAMDEC1 antibody, raised in rabbit at dilution of 1:30 for 60min at room temperature. Goat anti-rabbit poly-HRP-IgG was applied for 60min at room temperature followed by DAB enhancer solution for 10min. Slides were imaged with a Hamamatsu NanoZoomer 2.0-HT C9600 [Hamamatsu, Hertfordshire, UK].

### 2.5. Immunoblot

Mouse bowel was homogenised and lysed in Laemmli sample buffer containing β-mercaptoethanol [Sigma], protease inhibitors [Roche], and phosphatase inhibitors [Sigma]. Samples run on SDS-PAGE gels were transferred onto Hybond-P PVDF membranes [Amersham, Buckinghamshire, UK]. Membranes blocked in 5% non-fat milk were probed with anti-mouse ADAMDEC1 antibody and actin [Sigma A5060, overnight at 4ºC, followed by anti-rabbit IgG-HRP [Cell Signaling #7074] for 1h at room temperature.

*C. rodentium* protein lysates were blocked with bovine serum albumin [Sigma] followed by serum from *Adamdec1*^*+/+*^ and *Adamdec1*^*-/*-^ mice. Anti-mouse IgG-HRP was applied for 30min to detect anti-*C. rodentium* IgG.

Bound antibody was detected using ECL Plus [Amersham], exposed to Hyperfilm ECL [Amersham].

#### 2.5.1. Intestinal permeability

Mice were administered 600mg/kg of fluorescein isothiocyanate conjugated dextran [FITC-Dextran], MW 4 000 [Sigma Aldrich, FD4], dissolved in 200 µl PBS for gavage following a 3h fast. After 4h, serum samples were collected and serially diluted in PBS. The concentration of FITC in serum was determined by spectrophoto-fluorometry with an excitation of 485nm and an emission wavelength of 520nm using as standard serially diluted FITC-dextran. Serum from mice not administered FITC-dextran was used to determine the background.

#### 2.5.2. Cytokine assays

Mouse serum TNF, IL6, IL1β, IL10, IFNγ, and KC [CXCL1] levels were determined using the Mouse Proinflammatory Ultrasensitive plate [Meso Scale Discovery, Rockville, MD, USA] and read on a SECTOR^®^ Imager 6 000 [Meso Scale Discovery].

#### 2.5.3. Statistical analysis

Data are presented as mean ± standard error of the mean [SEM] using GraphPad Prism 4.03 [GraphPad Software, Inc.]. Statistical significance was calculated using paired or unpaired two-tailed Student’s t test. Mean differences were considered significant when *P* < 0.05.

## 3. Results

### 3.1. Tissue expression of ADAMDEC1 is highly conserved across animal species

**Figure 1.**
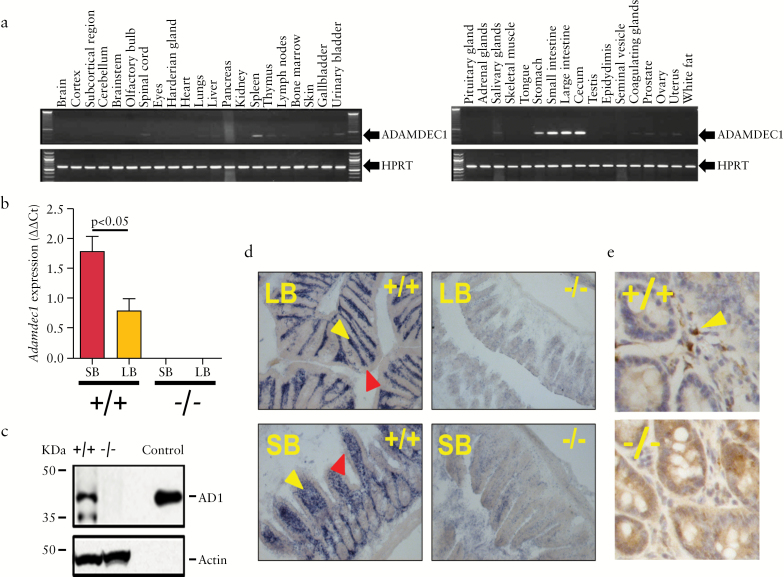
*Adamdec1* expression is restricted to the gastro-intestinal [GI ] tract. a. Tissue distribution of *Adamdec1* in the mouse. b. Quantitative PCR demonstrates that *Adamdec1* expression is higher in the small bowel [SB] that the large bowel [LB] of wild-type mice [+/+] relative to housekeeping gene *PPIA*. No *Adamdec1* expression was detectable in *Adamdec1^-/-^* mice [-/-]. c. Western blot analysis was used to investigate the presence of ADAMDEC1 protein in mouse colonic tissue; as expected *Adamdec1^-/-^* colonic tissue was devoid of protein. Actin was used as a loading control and recombinant mouse ADAMDEC1 [control] was run to confirm the specificity of the antibody. d. *In situ* hybridisation was performed on large [LB] and small [SB] bowel tissue from wild-type and *Adamdec1^-/-^* mice in order to ascertain the location of the mRNA expression. *Adamdec1*-expressing cells [blue stain] were restricted to the lamina propria region [yellow arrows], and the epithelium [red arrows] demonstrated no expression in either the large or the small bowel. There was no specific staining in the tissue obtained from the *Adamdec1^-/-^* animals. e. Immunohistochemistry revealed that the ADAMDEC1 protein was highly expressed [brown stain] in cells located in the lamina propria [yellow arrows] in mouse small bowel. Knockout tissue demonstrated no specific staining.

In the steady state in humans, expression of ADAMDEC1 is almost exclusively restricted to the macrophages of the GI tract.^16^ The gene atlas database, BioGPS [http://biogps.org/#goto = welcome], revealed a conserved expression pattern for *Adamdec1* across numerous mammalian species including pigs and gorillas [data not shown]. A low level of expression has also been reported in activated dendritic cells, from secondary lymphoid organs, in humans and mice.^13,16^ Our results were in accordance with those previous descriptions. Using a panel of mouse tissues, *Adamdec1* gene expression was shown to be predominantly in the small intestine, caecum and large intestine [Figure 1a]. A similar expression pattern was also seen in humans [Supplementary Figure 1a, available as Supplementary data at *ECCO-JCC* online]. *Adamdec1* expression was significantly higher in the small intestine compared with the colon in both mice [Figure 1b] and endoscopic pinch biopsies from healthy human volunteers [Supplementary Figure 1b].

Western blots on colonic lysates from wild-type mice identified two distinct bands that were absent from *Adamdec1*^*-/*-^ mice [Figure 1c]. The higher molecular weight band [~ 42 kDa] migrated in an identical manner to a recombinant full-length mouse ADAMDEC1-His tagged protein. The lower molecular weight band was roughly the same size, ~ 35kDa, as previously reported for active ADAMDEC1 protein.^13^

*In situ* hybridisation [Figure 1d] and immunohistochemistry [Figure 1e] localised the cells expressing or containing ADAMDEC1 in the large and small bowel to the lamina propria. These findings were replicated in human colonic tissue [Supplementary Figure 1c, d]. In both mice and humans the ADAMDEC1-positive cells in the intestine were mononuclear and consistent with intestinal macrophages by microscopy [Figure 1e]. Intestinal epithelial cells were not shown to express ADAMDEC1 in either mice [Figure 1d, e] or humans [Supplementary Figure 1c, d].

### 3.2. ADAMDEC1 expression does not require the presence of gut microflora

**Figure 2.**
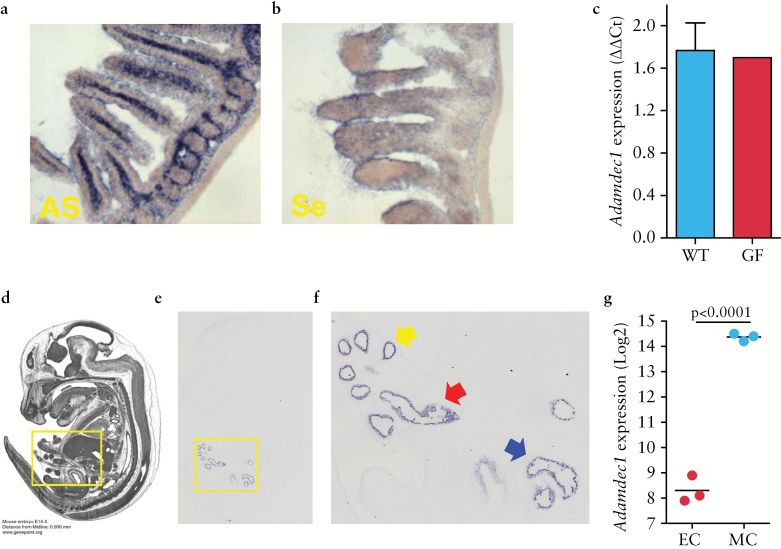
The expression of *Adamdec1* is not dependent on the presence of the gut microbiota. a. *In situ* hybridisation using an anti-sense probe [AS] for *Adamdec1* was performed on small bowel sections from wild-type GF mice [20 x magnification]. The presence of *Adamdec1*-specific mRNA was confirmed in this tissue [blue] and the distribution was identical to that seen in the conventional C57BL/6 wild-type mice [Figure 1d]. b. *In situ* hybridisation using the control sense-strand probe [Se] for *Adamdec1* demonstrates minimal non-specific binding to the small bowel section from the GF mice. c. Equivalent expression of *Adamdec1* was seen in wild-type and GF tissue using qPCR. Images d-f are taken from [http://www.genepaint.org, Genepaint Set ID: EG337] and represent, d, an MRI section image of an E14.5 C57BL/6 embryo sectioned through the midline. e. A corresponding section was used for *in situ* hybridisation of *Adamdec1* [5 x magnification]. The yellow box denotes the area of positive *Adamdec1* expression. f. This region is shown at an increased magnification. The *Adamdec1*-expressing tissue corresponds to the foregut [yellow arrow], midgut [red arrow], and hindgut [blue arrow]. g. *Adamdec1* is highly expressed in mesenchymal compared with epithelial cells in the embryological gut at E18.5, microarray data set [GSE 6383].

Due to the restricted tissue expression of ADAMDEC1 to regions of the bowel with a high concentration of microorganisms, and the increased expression observed in monocyte-derived macrophages [MDM] after exposure to LPS, it has been proposed that the gut microflora may be responsible for stimulating the induction of this gene.^16^ To test this theory, we measured the expression of *Adamdec1* in germfree (GF) mice. mRNA expression levels were equivalent in adult germ-free and wild-type animals [Figure 2a, c], indicating that it was not dependent on the presence of gut microflora.

Further evidence to support the presence of *Adamdec1* in the germ-free gut was found in two online datasets. An online embryonic mouse gene expression atlas resource [http://www.emouseatlas.org/emap/home.html] demonstrated that *Adamdec1* was expressed in the developing intestine at embryonic E14.5, before bacteria colonise the gut [Figure 2d-f]. Extrapolation of *Adamdec1* gene expression from transcriptomic profiles of mesenchymal and epithelial cells isolated from the small intestine of E18.5 mice confirmed the presence of *Adamdec1* in the prenatal sterile gut. Significantly higher levels of *Adamdec1* were seen in embryonic intestinal mesenchymal cells compared with epithelial cells, similar to humans and adult mice [Figure 2g]. These findings provide supportive evidence that the presence of *Adamdec1* in the gut is not solely dependent on a microbial stimulus.

### 3.3. *Adamdec1*^*-/*-^ mice do not present a spontaneous adverse phenotype

Global gene deletion of *Adamdec1* on a murine C57BL/6 background resulted in the complete loss of expression at both the mRNA and protein levels [Figure 1b-e]. Loss of *Adamdec1* had no obvious impact on the health of mice maintained in specific pathogen-free [SPF] conditions. *Adamdec1*^*-/*-^ mice were fertile, born in expected Mendelian ratios [M:F 1:1.1, *n* = 179, *P* = 0.5] and displayed no abnormal developmental phenotype or growth impairment [Supplementary Figure 2b, available as Supplementary data at *ECCO-JCC* online]. Histological assessment of the GI tract [Supplementary Figure 2a] and FACS analysis of the colonic leukocyte populations [Figure 4d] revealed no gross abnormality in *Adamdec1*-deficient mice. Loss of *Adamdec1* had no significant effect on intestinal permeability in the naïve, non-inflamed state [Supplementary Figure 2c]. Knockout mice were maintained in SPF conditions for up to 1 year without the development of spontaneous colitis or any other adverse phenotype. A recent publication by Vanden Berghe *et al.* identified passenger mutations in knockout mice generated through the use of 129 and C57BL/6J congenic mice.^19^ Their online software program identified two single nucleotide polymorphisms, close to the *Adamdec1* gene, affecting the coding sequences of *Tnfsf10b* and *Pnma2* genes, which had a > 90% chance of being retained in the *Adamdec1*^*-/*-^ mice. Neither of these genes are, however, expressed in the colon of wild-type mice either in the naïve state or during a dextran sodium sulphate [DSS]-induced colitis [data not shown]. It is therefore unlikely that these passenger mutations, if present, would have any significant bearing on our studies of gut inflammation in *Adamdec1*^*-/*-^ and wild-type mice.

### 3.4. *Adamdec1* is upregulated in the intestine during DSS-induced colitis and loss of expression results in an increased systemic response

**Figure 3.**
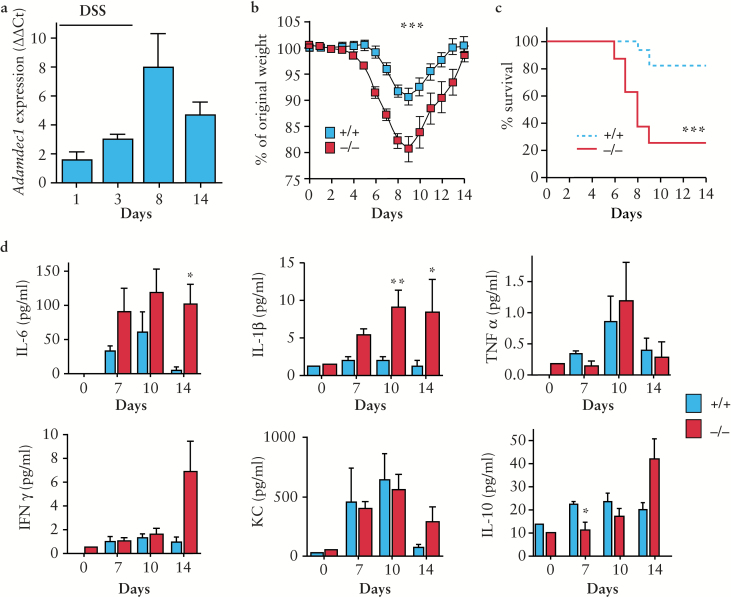
*Adamdec1* is up-regulated in the intestine during a chemical induced colitis. Loss of *Adamdec1* results in an increased systemic response to dextran sodium sulphate [DSS]. *Adamdec1*^*-/*-^ and *Adamdec1*^*+/+*^ [wild-type] mice were exposed to 7 days of 2% DSS. a. Quantitative PCR of *Adamdec1* relative to housekeeping gene *Ppia* in the colon of wild-type mice culled at set time points during and after the DSS challenge [*n* = 3–4 at each time point]. b. The change in original body weight and c. survival curves show that *Adamdec1*^*-/*-^ mice experience significantly greater weight loss and a higher rate of mortality, as defined as loss of > 15% of body weight, than *Adamdec1*^*+/+*^ mice following exposure to DSS [combination of four experiments, *n* = 25 mice per genotype]. d. Serum IL-6, IL-1β, TNF, INFγ, KC [IL-8], and IL-10 were measured in naïve mice, Days 7 and 10, and 14 days after DSS, in tail bleed and cardiac puncture serum [*n* = 5 mice per group, these results have been verified in a second cohort, data not shown]. A significant increase in the serum cytokines IL-1β and IL-6 is seen by Days 10 and 14, whereas IL-10 was lower in *Adamdec1*^*-/*-^ compared with *Adamdec1*^*+/+*^ *mice.* Results shown are mean ± standard error of the mean [SEM]. [*p < 0.05, **p < 0.01, and ***p < 0.001; two-tailed, unpaired *t* test.]

*Adamdec1*^*-/*-^ and wild-type mice were exposed to the colitogenic agent DSS [2% in the drinking water] for 7 days. In wild-type mice, *Adamdec1* was significantly upregulated in the colon during exposure to DSS, and on withdrawal of DSS *Adamdec1* levels decreased towards baseline, mirroring the inflammatory response [Figure 3a]. Following DSS challenge, *Adamdec1*^*-/*-^ mice demonstrated an earlier and more pronounced systemic response than wild-type counterparts, with significantly greater weight loss [Figure 3b] and a higher mortality rate [Figure 3c]. Mice lacking *Adamdec1* expression lost on average 20% of their original body weight and 80% died by Day 9 as compared with the wild-type mice which lost on average only 10% of their body weight and 20% died. These results clearly show that *Adamdec1* deficiency renders mice more susceptible to DSS-induced colitis. Mice heterozygous for *Adamdec1* demonstrated a response between that of the homozygous knockout and that of the wild-type mice [Supplementary Figure 3a, b, available as Supplementary data at *ECCO-JCC* online].

Exposure to DSS for 7 days resulted in increased serum levels of IL-6, IL-1β, TNF, KC, IFNγ, and IL-10 in wild-type and *Adamdec1*^*-/*-^ mice compared with naïve animals [Figure 3d]. Serum levels of IL-6 and IL-1β were significantly elevated in *Adamdec1*^*-/*-^ mice on Days 10 and 14 above those of the wild-type mice. In contrast, the levels of IL-10 were reduced in the knockout animals compared with the wild type. The circulatory levels of TNF, IFNγ, and KC were similar in both genotypes.

### 3.5. Loss of *Adamdec1* expression results in an increased sensitivity to DSS-induced colitis, with an elevation in neutrophil recruitment to the colon and increased expression of colonic IL-22 and IL-17

**Figure 4.**
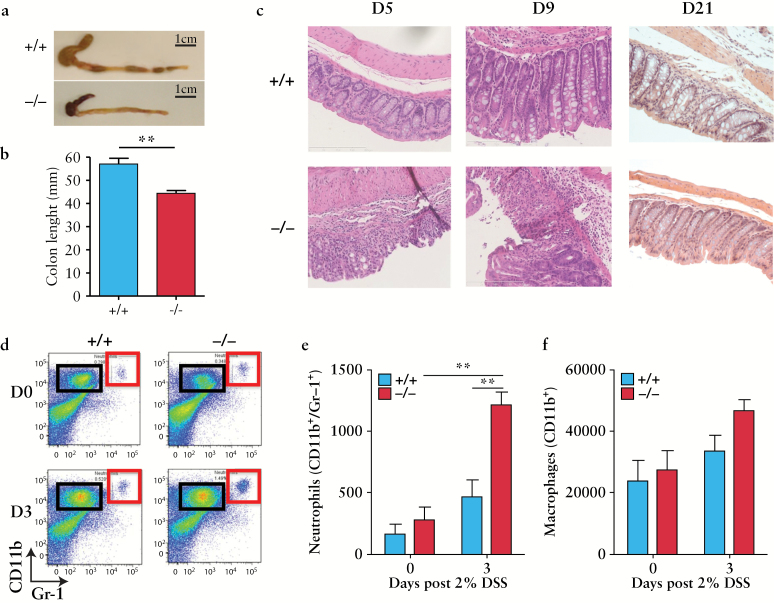
Loss of *Adamdec1* results in an increased susceptibility to dextran sodium sulphate [DSS]-induced colitis. a. Representative photographs of colons resected post mortem [caecum attached] from *Adamdec1* knockout [-/-] and wild-type [+/+] mice on Day 9 post DSS. The colons from *Adamdec1*^*-/*-^ mice exposed to 7 days of 2% DSS contained bloody stools, seen here in the shrunken caecum, and were significantly shorter than those from wild-type mice. b. Colon lengths on Day 9 [*n* = 7 per genotype]. c. Representative image of haematoxylin and eosin [H&E]-stained large bowel tissue [20 × magnification, scale bar: 200  µm] on Days 5, 9, and 21 after the DSS challenge demonstrate a more severe colitis in the *Adamdec1*^*-/*-^ mice with increased cellular infiltration, crypt distortion on Days 5 and 9, and ulceration on Day 9 in the knockout mice. On Day 21, the colons of the surviving *Adamdec1*^*-/*-^ and wild-type mice showed resolution of inflammation with normal tissue architecture and similar histological appearances on H&E staining. d. Representative FACS plots of isolated colonic lamina propria cells in naïve mice [D0] and 3 days post DSS [D3] in *Adamdec1* knockout [-/-] and wild-type [+/+] mice. The proportion of neutrophils [CD11b^+^/Gr-1^+^, red box] and macrophages [CD11b^+^/Gr-1^-^, black box] were determined using FACS analysis. e. The levels of tissue-resident macrophages [CD11b^+^/Gr-1^-^] and neutrophils [CD11b^+^/Gr-1^+^] are similar between the naive wild-type and *Adamdec1*^*-/*-^ mice [Day 0]. An increase in tissue-resident neutrophils, corrected for cell numbers, was observed in knockout animals exposed to DSS for 3 days. Wild-type animals demonstrated no significant increase in neutrophil numbers by Day 3. f. The number of tissue- resident macrophages, corrected for cell numbers, did not significantly change after 3 days of DSS exposure in either the knockout or the wild-type animals. Results are expressed as the mean ± standard error of the mean [SEM]. [*p < 0.05, **p < 0.01.]

The increased sensitivity of mice lacking *Adamdec1* to DSS resulted in more pronounced phenotypic changes to the colonic tissue [Figure 4 a-c]. There was a significant shortening of the colon in wild-type and *Adamdec1*^*-/*-^ mice 9 days post initiation of DSS, but this was more marked in the knockout animals [Figure 4a, b]. Histological examination of the colon revealed that the colitis was more severe in the knockout mice than the wild type on Days 5 and 9 [Figure 4c]. Mice that survived the acute DSS-induced colitis demonstrated restoration of normal colonic architecture by Day 21, irrespective of genotype [Figure 4c]. These results suggest that *Adamdec1* plays an active part in the initial inflammatory response to DSS, but has a negligible role in the resolution phase after removal of the insult.

FACS analysis of colonic leukocyte populations was performed pre and post DSS exposure in *Adamdec1*^*-/*-^ and wild-type mice [Figure 4d-f, Supplementary Figure 4, available as Supplementary data at *ECCO-JCC* online]. There was an elevation in neutrophils [CD11b^+^Gr-1^+^] in the *Adamdec1*^*-/*-^ compared with wild-type mice after 3 days of DSS exposure [Figure 4d, e], demonstrating that the *Adamdec1*-deficient animals develop an earlier and more aggressive inflammatory response. No significant difference in the number of macrophages [CD11b^+^ Gr-1^-^] [Figure 4d, f], dendritic [CD11c^+^ CD11b^-^] [Supplementary Figure 4a, b], or CD3+ T [data not shown] cells was observed; individual T cell subsets were not analysed in this study.

Colonic expressions of TGF-β, IL-22, and IL-17 were analysed following DSS exposure. No difference in TGF-β was observed throughout the course of DSS [Supplementary Figure 4c]. However, expressions of IL-22 and IL-17 were exaggerated in the knockout mice during DSS-induced inflammation. A rapid increase in IL-22 expression was noted in the *Adamdec1*^*-/*-^ mice compared with wild-type mice, as early as Day 3; both IL-22 and IL-17 were significantly raised in knock out mice on Day 15 and returned to baseline wild-type levels on resolution of inflammation by Day 21.

### 3.6. *Adamdec1*^*-/*-^ mice are more susceptible to *C. rodentium*-induced colitis

**Figure 5.**
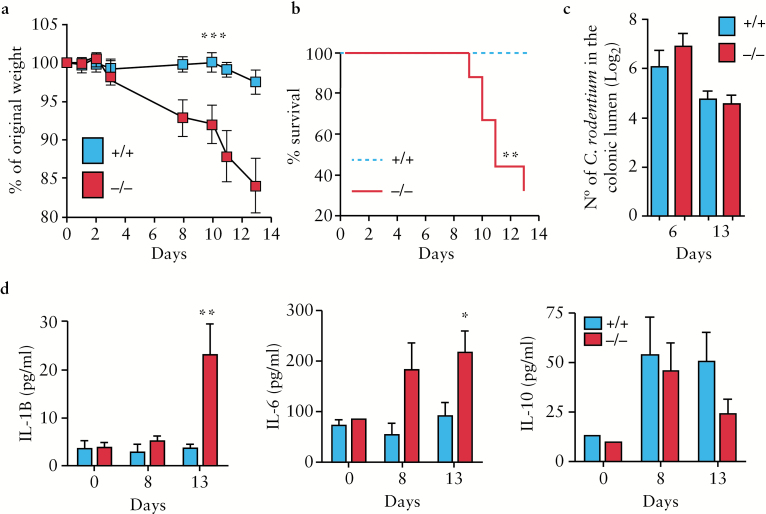
*Adamdec1* deficiency results in an increased susceptibility to *C. rodentium.* a. *Adamdec1*^*-/*-^ mice demonstrate a significant reduction in total body weight from Days 8 to 13 after *C. rodentium* inoculation [~ 10^9^] compared with wild-type animals [*n* = 15 per genotype, results replicated in four further experiments, data not shown]. b. An increased level of mortality was evident in *Adamdec1*^*-/*-^ mice compared with wild-type animals. c. The levels of *C. rodentium* measured in faecal samples were similar between *Adamdec1*^*-/*-^ and wild-type mice as shown on Days 6 and 13. d. IL1β is significantly elevated in the serum of *Adamdec1*^*-/*-^ mice on Day 13 post infection compared with naïve and wild-type animals. IL-6 is significantly elevated in the serum of *Adamdec1*^*-/*-^ mice on Day 13 post infection compared with naïve and wild-type animals. IL10 is elevated in both *Adamdec1*^*-/*-^ and wild-type mice compared with naïve animals, but no difference is observed between genotypes [*n* = 4 per genotype, replicated in second experiment, data not shown]. Results are expressed as the mean ± standard error of the mean [SEM]. [*p < 0.05, **p < 0.01, ***p < 0.001.]

To investigate the potential role of *Adamdec1* in bacterial-induced colitis we used the Gram-negative enteric bacterium, *C. rodentium,* which is capable of inducing a self-limiting colitis in mice.^20^ Following inoculation with ~ 10^9^ organisms, *Adamdec1*^*-/*-^ mice demonstrated significantly greater weight loss [Figure 5a] and mortality [Figure 5b] than the wild type. Wild-type mice exposed to *C. rodentium* did not demonstrate a significant change in body weight over the duration of the assay, and no animals succumbed to the infection. By contrast, animals lacking *Adamdec1* lost approximately 15% of their starting weight and 70% died over the 13-day period. Heterozygous [*Adamdec1*^*+/*-^] mice responded in a similar way to the wild type [data not shown]. The administration of a 10-times lower inoculation dose of *C. rodentium* [10^8^] induced a significant loss of weight in *Adamdec1*-deficient animals, whereas the wild-type mice continued to gain weight over the same period [Supplementary Figure 3c]. There was no mortality in either the wild-type or knockout animals at this lower inoculation dose [Supplementary Figure 3d]. The numbers of *C. rodentium* in the faeces were similar in both genotypes [Figure 5c]. These results demonstrate an increased sensitivity to *C. rodentium* infection in animals lacking *Adamdec1* expression, despite adequate intraluminal clearance.

### 3.7. *Adamdec1*^*-/*-^ mice are more susceptible to colonic inflammation and systemic infection upon *C. rodentium* infection

Following *C. rodentium* inoculation, serum levels of IL-6, IL-1β, TNF, KC, IFNγ, and IL-10 were measured in *Adamdec1*^*-/*-^ and wild-type mice. The serum levels of both IL-1β and IL-6 were significantly higher in *Adamdec1*-deficient as compared with wild-type mice by Day 13 [Figure 5d]. IL-10, TNF, IL12p40, and KC serum levels were similar in both genotypes [Figure 5d and data not shown].

**Figure 6.**
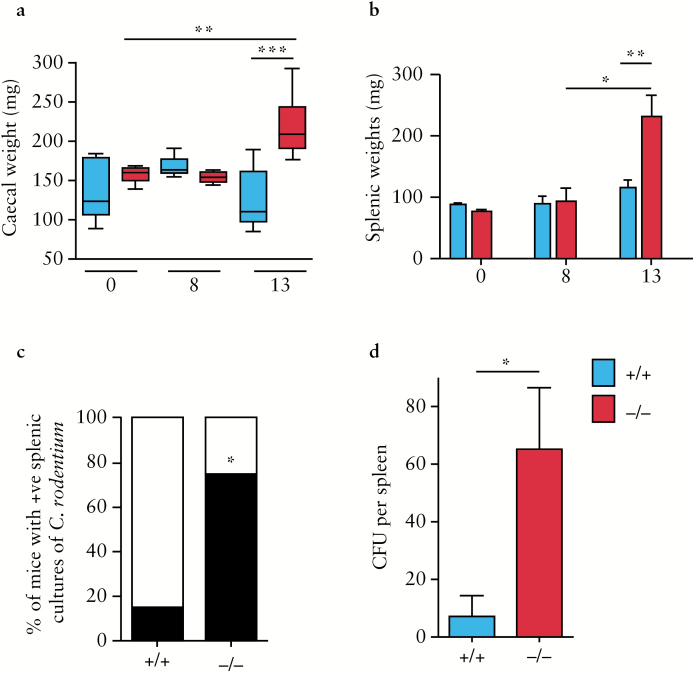
Increased susceptibility of *Adamdec1*^*-/*-^ mice to *C. rodentium* is associated with an elevation in tissue inflammation and systemic infection. a. Change in caecal weight post *C. rodentium* infection. *Adamdec1*^*-/*-^ mice demonstrate an increase in total caecal weight 13 days post infection. No alteration in caecal weight is evident in the wild-type mice. b. Change in splenic weight post *C. rodentium* infection. *Adamdec1*^*-/*-^ mice demonstrate an increase in splenic weight 13 days post infection. No alteration in splenic weight is evident in the wild-type mice. c. Percentage of mice with live *C. rodentium* in their spleens 13 days post infection (Black bar). *Adamdec1*^*-/*-^ mice were found to have live *C. rodentium* in their spleens more frequently than wild-type animals. d. A higher concentration of *C. rodentium* [colony-forming units, CFU] was found in spleens from *Adamdec1*^*-/*-^ mice compared with wild-type animals. Results are expressed as the mean ± standard error of the mean [SEM]. [*p < 0.05, **p < 0.01, ***p < 0.001.]

*C. rodentium* infection induces a colitis of the caecum and colon in mice. In *Adamdec1*^*-/*-^ mice, a significant increase in the total weight of the caecum was observed at 13 days post inoculation, suggesting an increased inflammatory infiltration. In comparison, wild-type mice did not demonstrate a significant change in caecal mass [Figure 6a].

Increased sensitivity to *C. rodentium* in mice lacking *Adamdec1* may result in an increase in systemic infection. In order to test this, spleens were isolated, weighed, and screened for the presence of live *C. rodentium*. The splenic weight did not alter during *C. rodentium* infection in wild-type animals, whereas a significant increase in splenic mass was observed in *Adamdec1*^*-/*-^ mice by Day 13 [Figure 5b]. The increase in mass coincided with higher numbers of live *C. rodentium* in the spleens of *Adamdec1*^*-/*-^, compared with wild-type, mice [Figure 5c, d]. These findings are consistent with impaired containment of the bacteria locally in the bowel and increased bacterial translocation into the circulation in mice lacking *Adamdec1*.

It has been reported that clearance of *C. rodentium* from the gut is dependent on B cells and IgG secretion.^21^ *Adamdec1*-deficient mice are however capable of mounting an antibody response to *C. rodentium* infection. Antibodies against *C. rodentium* protein lysate were detectable by Day 13 in the serum from *Adamdec1*^*+/+*^ and *Adamdec1*^*-/*-^ mice [Supplementary figure 5, available as Supplementary data at *ECCO-JCC* online]. These results demonstrate that *Adamdec1* deficiency does not influence the development of a B cell-mediated adaptive immune response to *C. rodentium* infection.

### 3.8. Adamdec1^-/-^ mice are more susceptible to *S. typhimurium* infection

**Figure 7.**
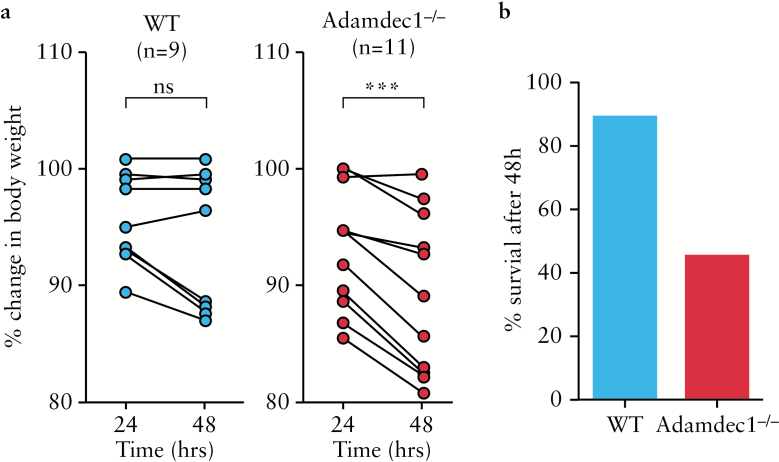
Increased susceptibility of *Adamdec1*^*-/*-^ mice to *S. typhimurium* infection. a. Percentage change in original body weight between 24 and 48h post oral infection with *S. typhimurium*. b. Percentage of animals surviving after 48h of infection with *S. typhimurium.* Paired Students *t* test, ***p < 0.001; ns = non-significant.

*S. typhimurium* infection in mice results in a transient systemic infection and fever, controlled initially through the activation of the innate immune system, followed by generation of an adaptive response.^22^ The major site of infection is in the small intestine where access to the host occurs through M cells within the epithelium. Pre-treatment of mice with antibiotic allows colonisation of the intestine and development of colitis. To assess the role of *Adamdec1* in protecting the mouse from *S. typhimurium* infection, antibiotic pre-treated knockout and wild-type mice were orally gavaged with 10^8^ live organisms and monitored for 48h [Figure 7]. *Adamdec1*^*-/*-^ mice lost a greater proportion of their body weight after *S. typhimurium* inoculation compared with the wild type [Figure 7a] and, at 48h post infection, 55% of the knockout compared with only 11% of the wild-type mice had succumbed to the infection [Figure 7b]. These results suggest that *Adamdec1* provides some protection to the host during the early phase of *S. typhimurium* infection.

## 4. Discussion

ADAMDEC1 is a unique member of the classical ADAM family,^13^ a group of proteins recognised as critical players in immunity and tissue remodelling.^23^ In the steady state, we have shown that ADAMDEC1 is abundantly and almost exclusively expressed in the GI tract, particularly in the small bowel. A highly conserved tissue expression pattern coupled with an increased susceptibility to GI infection upon loss of this protein demonstrates that ADAMDEC1 is an important component of the GI immune system.

In the intestinal wall we have demonstrated that ADAMDEC1 is expressed predominantly in mononuclear cells within the lamina propria in mice and humans. ADAMDEC1 has previously been isolated from human colonic macrophages; and extrapolation of ADAMDEC1 expression profiles, from online datasets of isolated murine colonic lamina propria cells, supports the finding of ADAMDEC1 in resident intestinal macrophage populations, in the steady state [GSE27859; GSE42101; GDS2982].^16,24–26,^ Classically these CD45^+^CD11b^+^F480^+^CD11c^+/-^Ly6c^hi^CD103^-^CX3CR1^hi^ intestinal lamina propria cells are reported as functionally phagocytic, bactericidal, non-migratory, tolerant, and derived from peripheral blood monocytes.^27–29^ In addition, peripheral blood monocytes cultured under sterile conditions *in vitro* upregulate ADAMDEC1 expression as they differentiate into macrophages.^8,16^ ADAMDEC1 is not expressed in resident macrophages from extra-intestinal tissues. A number of studies and online datasets demonstrate that ADAMDEC1 is not detectable in mature macrophage populations resident in the peritoneum, lung, liver, and adipose tissues and is only moderately expressed in mature splenic macrophages [GSE56711; GDS2982].^24,30^ However, during inflammation ADAMDEC1 has been reported to be upregulated at sites where it is not constitutively expressed, such as lung, joints, and major blood vessels in pulmonary sarcoidosis,^4^ osteoarthritis,^5^ and atherosclerotic plaque instability,^3^ respectively. This association with human inflammatory diseases has led to speculation that ADAMDEC1 may play a role in the human immune system and acute inflammatory response.^13,16^

At a cellular level, we and other investigators have demonstrated a rapid and robust induction of ADAMDEC1 in response to bacterial antigens, in particular LPS,^16^ which would support a role for ADAMDEC1 as a bacterial response gene. It has therefore been proposed that ADAMDEC1 expression is a consequence of background priming by LPS in the gut lamina propria.^13^ Our results do not support this supposition; we have presented evidence that ADAMDEC1 is also expressed in the GI tract of germ-free mice and in the un-colonised sterile prenatal embryological bowel. In addition, during MDM differentiation a significant elevation in ADAMDEC1 expression is observed in the absence of any bacterial ligands. These findings suggest that exposure to bacterial antigens is not a prerequisite for ADAMDEC1 expression in MDM or gut.

An alternative explanation for the tissue and cellular expression of ADAMDEC1 may be found in the origin of resident tissue macrophages. Historically, all tissue-resident macrophages were believed to be part of a linear mononuclear phagocytic system and originate from circulating blood monocytes, similar to inflammatory macrophages.^31^ There is, however, a growing body of evidence that resident macrophages in the mouse are laid down early in development, arise from embryonic progenitors^32^ in the yolk sac and or foetal liver, and undergo clonal expansion *in situ* with little or no contribution from circulating monocytes in adult life. The intestine is an exception to this rule and it has recently been shown that the resident macrophage population within the adult bowel is maintained through continual recruitment of circulating monocytes which originate from the bone marrow.^33^ These findings, coupled with *in vitro* data demonstrating that ADAMDEC1 expression is induced during peripheral blood monocyte to macrophage differentiation, suggest that these intestinal macrophages may differ from extra-intestinal yolk sac- and foetal liver-derived macrophages.^16^ It is probable therefore that the reason ADAMDEC1 is selectively expressed in the bowel is because it is the predominant tissue in which resident macrophages are derived from the peripheral blood monocyte pool in the naïve non-inflamed state. The reported expression of ADAMDEC1 in extra-intestinal diseased tissue could result from induction of ADAMDEC1 in MDM recruited to sites of inflammation.

In contrast to extra-intestinal tissues, ADAMDEC1 has been identified as significantly under-expressed in a number of diseases that affect the gut, such as GI cancers and Crohn’s disease.^7–9,11,12^ This phenomenon is unlikely to be secondary to bowel inflammation *per se*, as we have clearly shown that *Adamdec1* is significantly upregulated, at a tissue level, in the bowel of mice following exposure to a colitogenic agent and induction of an acute colitis. Furthermore, ADAMDEC1 has been reported as under-expressed in MDM and terminal ileal [TI] tissue from patients with quiescent as well as active Crohn’s disease.^7,8^ We predicted that loss of ADAMDEC1 may increase the host’s susceptibility to bacterial infection and intestinal inflammation resulting in the development of Crohn’s disease. This theory was substantiated through the use of a number of GI infection and inflammatory models in *Adamdec1*-deficient mice. These studies provided evidence supporting a role for ADAMDEC1 in the immune response of the gut. In pathogen-free conditions, the absence of *Adamdec1* expression has no noticeable effect on murine gut development, but subjecting *Adamdec1*-deficient mice to colitogenic agents revealed an elevated local and systemic inflammatory response, increased bacterial translocation and systemic infection, and greater mortality. It is plausible that ADAMDEC1 may also play an important role in human intestinal immunity and protection against the development of infection and chronic inflammation. These findings provide the first piece of evidence linking ADAMDEC1 to a regulatory role in bowel immunity.

The exact mechanism by which ADAMDEC1 exerts this protective effect against colitis is yet to be determined. ADAMDEC1 evolved from a superfamily of zinc-dependent proteolytic enzymes which are well recognised to activate and degrade a variety of substrates including chemokines, cytokines, growth factors, and extracellular matrix proteins.^2,23,34^ By their substrate interactions it has become increasingly evident that these metalloproteases influence the function and migration of inflammatory cells, maintain tissue homeostasis, aid wound healing, and are pivotal in the regulation of the innate and adaptive immune response. The proteolytic targets of ADAMDEC1, however, remain elusive. A number of metalloproteases have been shown to cleave, activate, and in some cases degrade IL-1β.^35–37^ *Adamdec1*^*-/*-^ mice display an exaggerated level of serum IL-1β and the downstream cytokine IL-6 during both chemical and bacterial induced colitis, which would suggest that *Adamdec1* may play a role in secretion and/or degradation of IL-1β. Both IL-1β and IL-6 are crucial for neutrophil recruitment to sites of infection and play a central role in preferentially inducing Th17 differentiation, independent of TGF-β.^38,39^ IL-6 has also been reported to contribute to innate immune-mediated chronic intestinal inflammation by promoting production of IL-22 and IL-17 by innate lymphoid cells [ILCs].^40^ Consistent with this finding, expressions of IL-22 and IL-17 were significantly increased in *Adamdec1*^*-/*-^ mice compared with wild-type animals, following exposure to DSS and induction of an experimental colitis, and no genotype difference was seen in TGF-β levels. Further study is required to ascertain the mechanism by which *Adamdec1* may target IL-1β and IL-6 and delineate the T cell and ILC response in *Adamdec1*^*-/*-^ mice.

A recent study has identified three other potential substrates [α2-macroglobulin, carboxymethylated transferrin, and casein] as well as demonstrating the lack of responsiveness to the classical ADAMs inhibitors TIMP1-3.^14,15^ It is unclear if the substrates identified so far are the true biological targets of ADAMDEC1, but it is plausible that ADAMDEC1 has evolved to escape inhibition by endogenous metalloprotease inhibitors. Further work is needed to identify the biologically relevant ligands for ADAMDEC1 and identify the mechanisms responsible for its expression and regulation within the gut and inflamed tissue.

## Funding

This work was supported by the Wellcome Trust [088683/Z/09/Z] and United Kingdom Medical Research Council [G0902005].

## Conflict of Interest

The authors have no conflicting financial interest.

## Author Contributions

NRO, TSC, JD, RM, BNS, PJS, SLB, AMS, and AWS planned and performed experiments. NRO, AMS, and AWS designed the studies and wrote the manuscript.

## Supplementary Data

Supplementary data are available at *ECCO-JCC* online.

Supplementary Figure 1a
