# Supplementary material for: Critical Role of the Disintegrin Metalloprotease ADAM-like Decysin-1 [ADAMDEC1] for Intestinal Immunity and Inflammation
Source: J Crohns Colitis. 2016 May 25;10(12):1417–27. doi: 10.1093/ecco-jcc/jjw111 (PMC5174729; doi:10.1093/ecco-jcc/jjw111)
Supplement: Supplementary Figure 1a [file ecco-jcc_jjw111_index.html]

Supplementary Data | Journal of Crohn's and Colitis

## Supplementary Data

Data files

- Supplementary Data - Supplementary Data
- Supplementary Data - Supplementary Data
- Supplementary Data - Supplementary Data
- Supplementary Data - Supplementary Data
- Supplementary Data - Supplementary Data
- Supplementary Data - Supplementary Data
