# Supplementary figures and images for: Critical Role of the Disintegrin Metalloprotease ADAM-like Decysin-1 [ADAMDEC1] for Intestinal Immunity and Inflammation
Source: J Crohns Colitis. 2016 May 25;10(12):1417–27. doi: 10.1093/ecco-jcc/jjw111 (PMC5174729; doi:10.1093/ecco-jcc/jjw111)

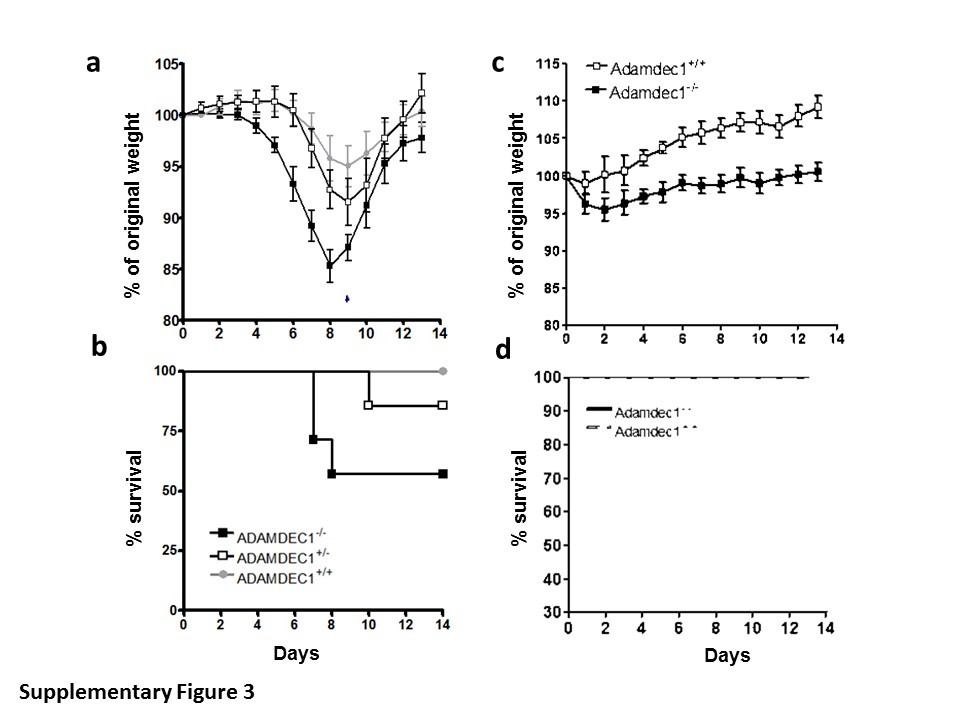

Supplement: Supplementary Figure 1a [file Supp3.jpg]

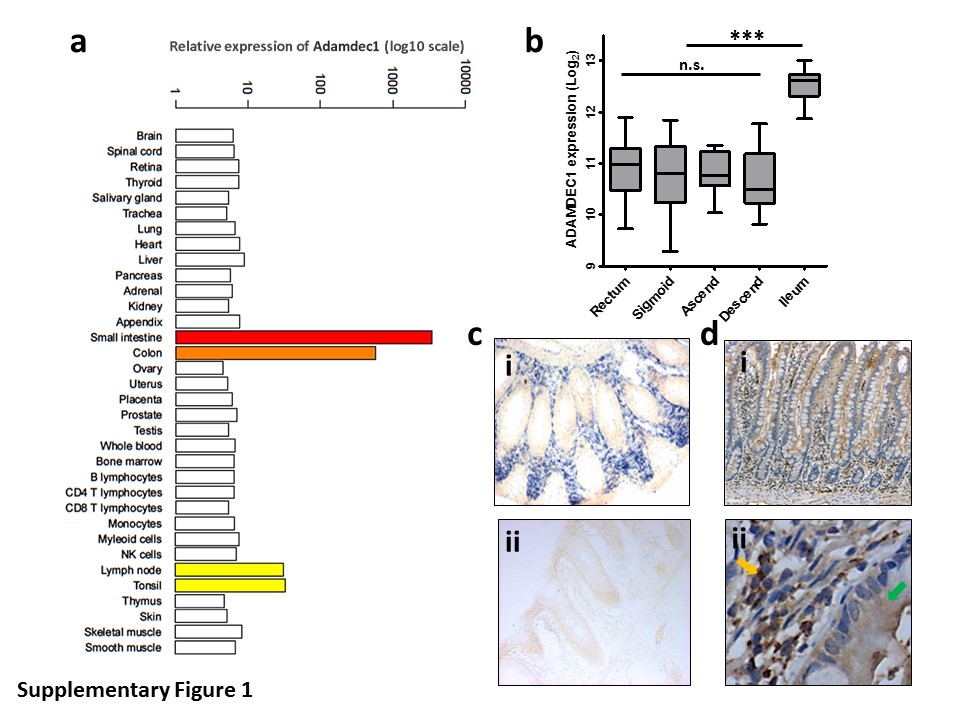

Supplement: Supplementary Figure 1a [file Supp1.jpg]

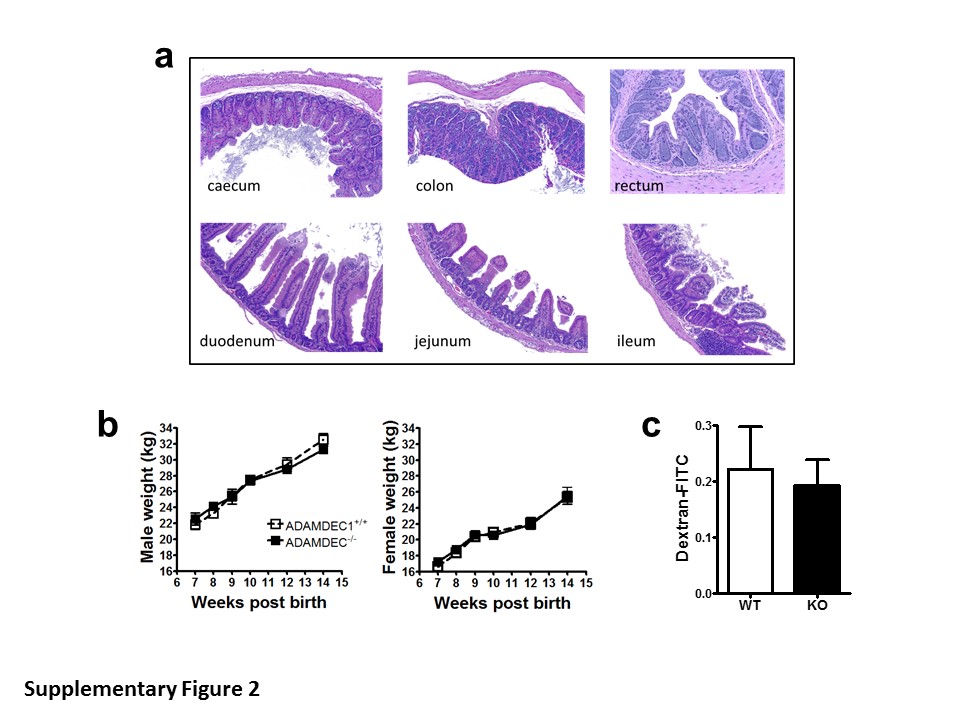

Supplement: Supplementary Figure 1a [file Supp2.jpg]

## Slide 1
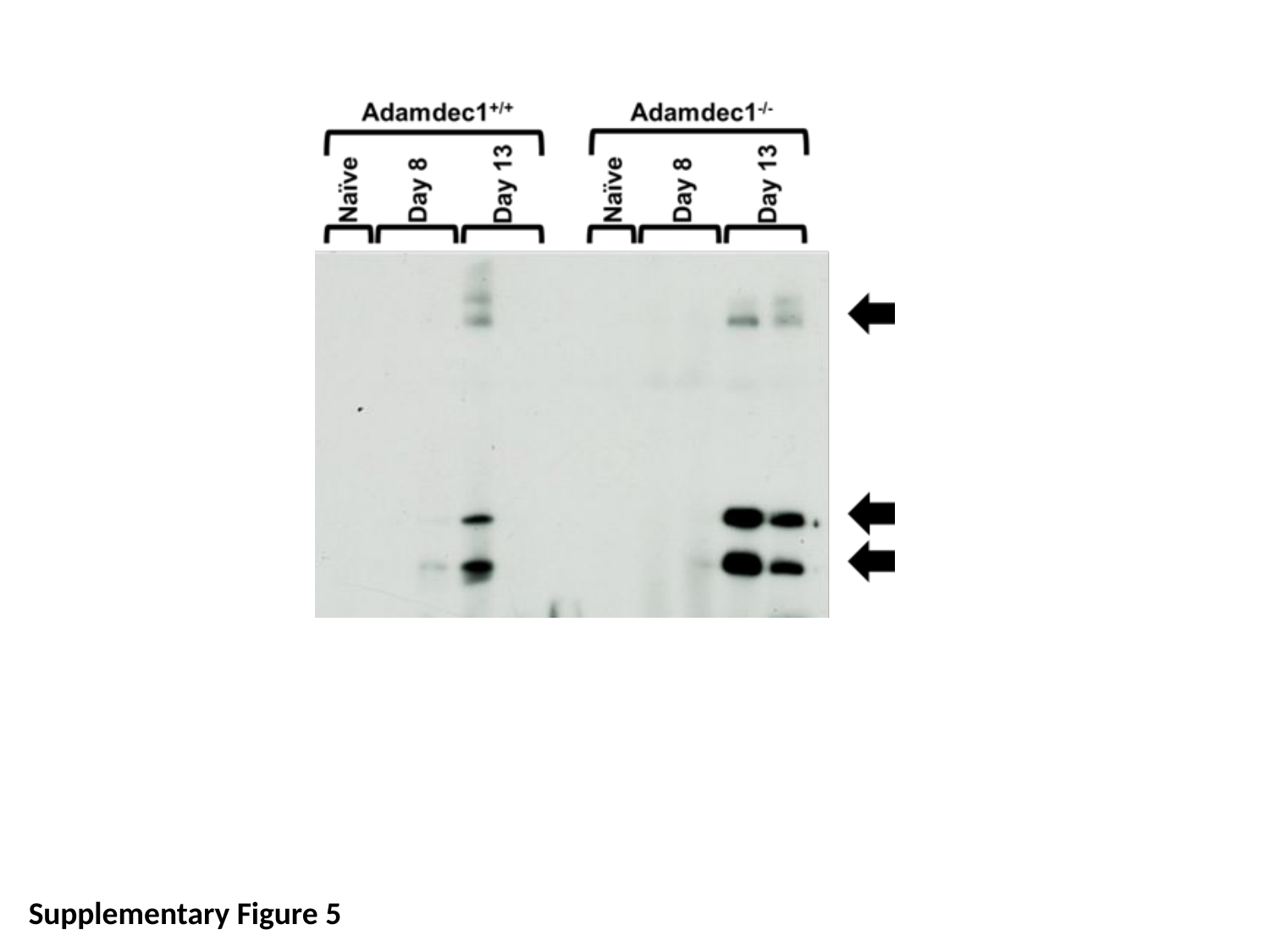

Supplementary Figure 5

Supplement: Supplementary Figure 1a [file Supp_fig_5.ppt]

## Slide 1
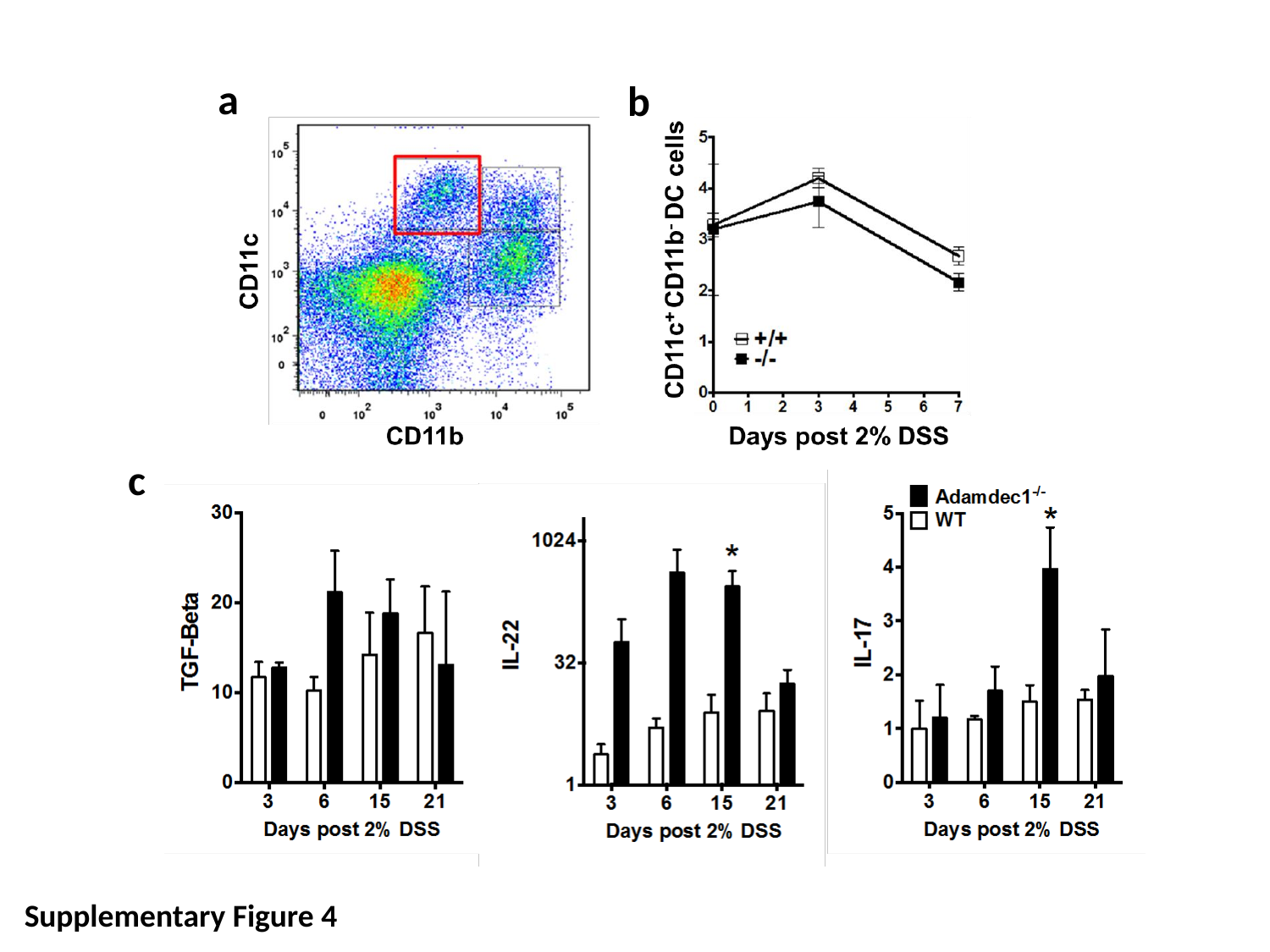

a
b
c
Supplementary Figure 4

Supplement: Supplementary Figure 1a [file Supp_fig_4.ppt]
