# Supplementary material for: Critical Role of the Disintegrin Metalloprotease ADAM-like Decysin-1 [ADAMDEC1] for Intestinal Immunity and Inflammation
Source: J Crohns Colitis. 2016 May 25;10(12):1417–27. doi: 10.1093/ecco-jcc/jjw111 (PMC5174729; doi:10.1093/ecco-jcc/jjw111)
Supplement: Supplementary Figure 1a [file Supplementary_figure_legends.docx]

**Supplementary figure legends:**

**Supplementary figure 1:** a. ADAMDEC1 is highly expressed in the human GI tract and to a lesser extent in the lymph node and tonsil, data extrapolated from the gene atlas data base, BIOGPS (Expression > log10^3^: red bars, >log10^2^: orange bars, >log10^1^: yellow bars, undetectable levels: white bars). b. ADAMDEC1 expression measured in human ileocolonic biopsies taken at endoscopy from healthy non inflamed controls confirm ADAMDEC1 is expressed at a significantly higher levels in the TI of the small bowel compared with rectum and colon (n=30 in colon, n= 10 in TI). Similar levels of expression of ADAMDEC1 were found in the rectum and throughout the colon. c. *In situ* hybridization was performed on colonic tissue from healthy surgical resection specimens from volunteers, (i) an antisense probe demonstrated mRNA expression of ADAMDEC1 was restricted to the lamina propria (blue), the epithelial cells were negative for ADAMDEC1, (ii) the control sense probe for ADAMDEC1 did not show binding. d. Immunohistochemistry revealed that the ADAMDEC1 protein was highly expressed (brown stain) in mononuclear cells located in the lamina propria (yellow arrows) in healthy human small bowel (i. x5 magnification, ii. X20 magnification). Results are expressed as the mean ± SEM *p<0.05, **p<0.01, p<0.001***, n.s. non-significant.

**Supplementary Figure 2.** a. Histology examination of the *Adamdec1^-/-^* mouse GI tract reveals no obvious developmental abnormalities. b. No evidence of a difference in growth rate of *Adamdec1^-/-^* mice in males and females (n=25 mice). c. Intestinal permeability measured by determining the concentration of FITC-Dextran (µg/ml) in the serum of *Adamdec1^+/+^* (WT) and *Adamdec1^-/-^* (KO) mice. Data are means ± SEM (n = 3), n.s. non-significant.

**Supplementary Figure 3.** a-b. 2% DSS colitis response of heterozygote *Adamdec1^+/-^* mice (open squares) compared to *Adamdec1*^-/-^ (black squares) and wild type animals (grey circles) (n=7 per genotype, representative of one of two experiments). c-d. *C. rodentium* infection demonstrates a dose dependent response in both *Adamdec1^-/-^* and wild type mice (n=10 per genotype). c. The increased susceptibility of *Adamdec1* deficient animals is evident at 10^8^ but *Adamdec1^-/-^* mice loose less weight following inoculation with 10^8^ *C. rodentium* compared with the higher dose of 10^9^ bacteria (fig 4). d. *Adamdec1* deficient animals do not succumb to *C. rodentium* infection at the lower dose of 10^8^.

**Supplementary Figure 4.**  a. Identification of colonic resident dendritic cells (CD11c^+^CD11b^-^) using multi-parameter FACS analysis and b. quantification of the dendritic cells, as a percentage of CD45^+^ cells, during a 2% DSS colitis in WT (open squares) and *Adamdec1^-/-^* (black squares) mice. c. Change in TGF-β, IL-22 and IL-17 mRNA levels within the colon during a 2% DSS colitis, relative to PPIA (ΔΔCt). For each genotype, n=3 mice per time point. Unpaired, two tailed, t-test *p<0.05. Results expressed as mean ±SEM.

**Supplementary Figure 5.** *Adamdec1* deficient mice are capable of mounting an antibody response to *C. rodentium* infection. Following oral inoculation, antibodies against *C. rodentium* protein lysate were detectable by day 13 in the serum from *Adamdec1^+/+^* and *Adamdec1^-/-^* mice (as highlighted by arrows).
